# Extrachromosomal Histone H2B Contributes to the Formation of the Abscission Site for Cell Division

**DOI:** 10.3390/cells8111391

**Published:** 2019-11-05

**Authors:** Laura Monteonofrio, Davide Valente, Cinzia Rinaldo, Silvia Soddu

**Affiliations:** 1 Unit of Cellular Networks and Molecular Therapeutic Targets, IRCCS-Regina Elena National Cancer Institute, 00144 Rome, Italy; davide.valente84@gmail.com (D.V.); cinzia.rinaldo@uniroma1.it (C.R.); 2 Institutes of Molecular Biology and Pathology (IBPM), National Research Council (CNR), c/o Sapienza University, 00185 Rome, Italy

**Keywords:** extrachromosomal histone H2B, abscission site, CHMP4B, ESCRT-III fission machinery

## Abstract

Histones are constitutive components of nucleosomes and key regulators of chromatin structure. We previously observed that an extrachromosomal histone H2B (ecH2B) localizes at the intercellular bridge (ICB) connecting the two daughter cells during cytokinesis independently of DNA and RNA. Here, we show that ecH2B binds and colocalizes with CHMP4B, a key component of the ESCRT-III machinery responsible for abscission, the final step of cell division. Abscission requires the formation of an abscission site at the ICB where the ESCRT-III complex organizes into narrowing cortical helices that drive the physical separation of sibling cells. ecH2B depletion does not prevent membrane cleavage rather results in abscission delay and accumulation of abnormally long and thin ICBs. In the absence of ecH2B, CHMP4B and other components of the fission machinery, such as IST1 and Spastin, are recruited to the ICB and localize at the midbody. However, in the late stage of abscission, these fission factors fail to re-localize at the periphery of the midbody and the abscission site fails to form. These results show that extrachromosomal activity of histone H2B is required in the formation of the abscission site and the proper localization of the fission machinery.

## 1. Introduction

Histones are the constitutive components of nucleosomes, the basic units of DNA packaging in eukaryotes, and their post-translational modifications regulate chromatin structure and activity. [1,2]. In mammals, there are different functional copies of the histone genes, which are located in a few clusters and encode several histone variants whose specific contribution to chromatin regulation is becoming clear [3,4,5,6,7]. In addition to chromatin structure and function, extrachromosomal activities of histones are emerging. In response to apoptotic stimuli, extrachromosomal histone H1.2 was shown to translocate into the cytoplasm and induce cytochrome C release from mitochondria [8] while histone H3 was shown to localize at the mitochondria [9]. In antiviral innate immune response, ecH2B was shown to contribute to sense DNA-virus genomes and induce the cytoplasmic IFN-β response [10,11]. In telomere homeostasis, a coding-independent role for histone mRNA was identified [12].

We previously identified an extrachromosomal localization of histone H2B at the intercellular bridge (ICB) during cytokinesis [13]. This H2B localization is independent of the presence of DNA, such as chromosome bridges, lagging chromatin, and ultra-fine BLM bridges at the cleavage plane [13], or RNA, such as microRNA, single-strand RNA, double-strand RNA, and RNA/DNA hybrid [14]. We have shown that ecH2B is recruited at the intercellular bridge by the Aurora-B kinase that binds and phosphorylates H2B at Ser32 [14]. In addition, ecH2B is phosphorylated at Ser14 by the Homeodomain interacting protein kinase 2 (HIPK2), a tyrosine-regulated kinase [15,16] required for faithful cytokinesis and prevention of tetraploidization, chromosomal instability, and tumorigenesis [13,17]. In particular, in HIPK2-defective cells, ecH2B is present at the ICB but is not phosphorylated at Ser14 [13]. However, the function exerted by ecH2B in cytokinesis is still unclear. 

Cytokinesis is the final stage of the cell cycle through which a mother cell divides into two daughter cells [18,19,20]. The process begins after chromosome segregation when the metaphase plane is specified and interpolar microtubules are bundled in an antiparallel manner to form the central spindle. With the cleavage furrow ingression the central spindle is narrowed to a thin ICB and its microtubules are compacted into a dense structure, the midbody. The midbody works as a platform for the specific spatiotemporal distribution of more than 150 proteins and lipids that contribute to abscission, when the ICB connecting sibling cells is physically separated [21,22,23,24,25]. This final event requires the definition of one or two abscission sites, also named the midbody side, secondary ingression site, or constriction site, where the physical separation takes place through coordinated membrane scission and microtubule severing [26,27]. Current models indicate that the endosomal sorting complex required for transport (ESCRT)-III machinery is the key component of the helical filaments that through contracting spirals and ring-like structures narrow the ICB at the abscission site to a point of fission [28,29,30,31,32,33]. The ESCRT-III complex consists of numerous charged multi-vesicular body proteins (CHMPs), which are first recruited at the midbody by the Centrosome Protein of 55 KDa (CEP55) and its downstream factors and then re-localized at the abscission site for the final cut together with the microtubule severing enzyme Spastin [23,27,34,35,36,37,38]. Recent evidence indicates that ESCRT-III re-localization has to be preceded by the generation and the proper maturation of the abscission site. This process is not yet fully understood but it has been shown to include remodeling of F-actin and other cytoskeletal filaments, such as septins and non-muscle myosin-II, whose inactivation cause abscission delay [30,39,40,41,42,43,44,45]. 

Here, we set out to elucidate the role of ecH2B at the ICB. Visualization of several cytokinesis factors in cells depleted of ecH2B demonstrated the contribution of this histone in the formation of the abscission site.

## 2. Materials and Methods

### 2.1. Cells, Culture Conditions, and Transfections

Human HeLa cells (kind gift of Dr. Claudio Passananti) and hTERT-immortalized dermal human fibroblasts (HFs; kind gift of Prof Fabrizio Loreni) were cultured in DMEM/GlutaMAX with 10% heat-inactivated fetal bovine serum (FBS; Life Technologies, Carlsbad, CA, USA) and maintained in a humid incubator at 37 °C in a 5% CO_2_ environment. Cells were routinely tested for mycoplasma contamination. For live cell imaging, HeLa cells were cultured in DMEM without phenol red, supplemented with 10% FBS. RNA interference was performed with commercially available H2B variant-specific stealth RNAi sequences (Life Technologies; Supplementary Table S1) and by universal negative control stealth RNAi, Negative Medium GC Duplexes (Life Technologies). Cells were transfected with a mix of nine siRNAs each at 10 nM or 16.5 nM in a double pulse, the second one 24 h after the first one by using RNAiMAX reagent (Life Technologies) according to the manufacturer’s instructions. Red fluorescent oligonucleotides (Block-it, Life Technologies) were used to evaluate transfection efficiency. GFP-H2B expression was obtained by cell transfection with the pBOS-GFP-H2B plasmid DNA (BD PharMingen, San Diego, CA, USA) and CHMP4B-MYC expression with the pCDNA5/FRT/TO (kind gift of Barbara Ciani) using Lipofectamine LTX and PLUS reagent according to the manufacturer’s instructions. 

### 2.2. Western Blotting 

Total cell lysates were prepared by using the following denaturing lysis buffer (50 mM Tris-HCl (pH 8), 600 mM NaCl, 0.5% sodium deoxycholate, 0.1% SDS, 1% NP40, and 1 mM EDTA) supplemented with protease-inhibitor mix (Roche, Basel, Switzerland) and Halt Phosphatase Inhibitor Cocktail (Life Technologies). Histone extraction protocol was performed following the Abcam protocol. Briefly, cells were harvested and washed twice with PBS. The cells were then resuspended in Triton Extraction Buffer (PBS containing 0.5% Triton X 100 (*v*/*v*), 2 mM PMSF, and 0.02% (*w*/*v*) NaN_3_) and lysed on ice for 10 min. The nuclei were isolated by centrifugation at 6500× *g* for 10 min at 4 °C and resuspended in 0.2 N HCl over night at 4 °C to extract histones. The supernatant (which contains the histone proteins) was neutralized with 2M NaOH at 1/10 of the volume of the supernatant. NuPAGE^®^ Novex Bis-Tris Gels (Life Technologies) were used for SDS-PAGE and nitrocellulose membranes (Bio-Rad Hercules, CA, USA) for protein transfer and immobilization. The following Abs were employed for WB: anti-α-tubulin moAb (Immunological Sciences, Rome, Italy), anti-GST moAb (kindly provided by Maurizio Fanciulli), anti-H2B moAb (Abcam, Cambridge, UK), HRP-conjugated goat anti-mouse, and anti-rabbit secondary Abs (Bio-Rad). Immunoreactivity was determined using the ECL-chemiluminescence reaction (AmershamCorp, Buckinghamshire, UK) following the manufacturer’s instructions.

### 2.3. Immunofluorescence Microscopy

Cells seeded on poly-l-lysine coated coverslips were fixed with 2% formaldehyde or ice-cold methanol, washed three times in phosphate buffered saline (PBS), permeabilized for 10 min with 0.25% Triton X-100 and blocked for 60 min with 5% BSA in PBS. Cells were stained with the Abs reported in Supplementary Table S2. Secondary FITC- and TRITC-conjugated Abs (Alexa-flour, Life Technologies) were used to detect mouse and rabbit primary Abs. DNA was marked with HOECHST 33342 (Sigma). Cells were examined with Olympus BX53 microscope equipped with epifluorescence and photographs were taken (×100 objective) using a cooled camera device (ProgRes MF, Jenoptik, Jena, Germany), with confocal microscope Zeiss LSM510-Meta, and LEICA inverted microscope DMi8 platform to measure midbody length with the application suite V4.7.

### 2.4. Live-Cell Imaging

Cells seeded on 15 μ-Slide 8 well (80826, ibiTreat, Ibidi, Gräfelfing, Germany) were observed under an Eclipse Ti inverted microscope using a Plan Apo 40× objective (Nikon). During the whole observation, cells were kept in a microscope stage incubator (Basic WJ, Okolab, San Bruno, CA, USA) at 37 °C and 5% CO_2_. DIC images were acquired every 3 min over a 24 hr period by using a DS-Qi1Mc camera and the NIS-Elements AR 3.22 software (Nikon, Tokyo, JP). Image and video processing were performed with NIS-Elements AR 3.22.

### 2.5. Proximity Ligation Assay

Cells seeded on round poly-L-lysine coated coverslips were processed for proximity ligation assay (PLA) using the Duolink^®^ In Situ Detection Reagents Red DUO92008 (Sigma-Aldrich, St. Louis, MO, USA) in four steps: (1) incubation of fixed cells with primary specific Abs; (2) incubation with secondary Abs conjugated with complementary oligonucleotide tails (PLA probes, called PLUS and MINUS); (3) ligase addition when, if the two proteins interact or are very close, the ligation step will produce a DNA circle; and (4) rolling circle amplification. Cells were fixed, blocked, and incubated with primary Abs as for IF; we used combination of mouse and rabbit primary Abs for each protein pair (rabbit anti-H2B-Ser14^P^ or -Ser32^P^ and mouse anti-CHMP4B). Anti-mouse MINUS and anti-rabbit PLUS PLA probes were added on coverslips (diluted 1:5 in PBS containing 0.05% Tween-20 and 3% bovine serum albumin) and incubated in a pre-heated humidity chamber (60 min at 37 °C). Subsequent ligation (30 min at 37 °C) and amplification (70 min at 37 °C) steps were performed following the protocol. To localize PLA signals, cells were fixed in formaldehyde 2% 10 min at RT and then co-stained using HOECHST 33342 and anti-alpha tubulin FITC-conjugated Ab.

### 2.6. In Vitro Binding Assay and H2B Phosphorylation

For H2B and CHMP4B binding assays, GST-CHMP4B (ag4544, Proteintech, Rosemont, IL, USA) was incubated overnight at room temperature with 500 ng of recombinant His-H2B (ag7811, Proteintech) or histone H2B (#14-491, Millipore) in buffer phosphate pH 7.5, 150 mM NaCl. GST-pulldown was performed by incubation for 2 h at 4 °C with Glutathione-Sepharose 4 Fast Flow beads (GE Healthcare, Buckinghamshire, UK) and three washes with buffer phosphate. Bound proteins were resolved by SDS-PAGE and analyzed by WB. For H2B phosphorylation, recombinant His-H2B was incubated with HIPK2 Kinase domain (kind gift of Dr. Linda Montemiglio), as an enzymatic source, in kinase buffer (Hepes 20 mM pH 7.5, 1 mM DTT, 10 mM MgCl2, and 1 mM EGTA) at 30 °C for 30 min in the presence of cold ATP or, as a control, of γ-^32^P-ATP (BLU502Z250UC, Perkin-Elmer, Waltham, MA, USA).

### 2.7. Statistics

Each experiment has been repeated from three to five times and the results obtained presented as mean ± standard deviation of the mean (SD). *p* values were derived from unpaired two-tailed *t*-tests using GraphPad Prism software (GraphPad Software, Inc., San Diego, CA, USA). *p* values < 0.05 were considered significant.

## 3. Results

### 3.1. Depletion of ecH2B Induces Accumulation of Long ICBs

**Figure 1:**
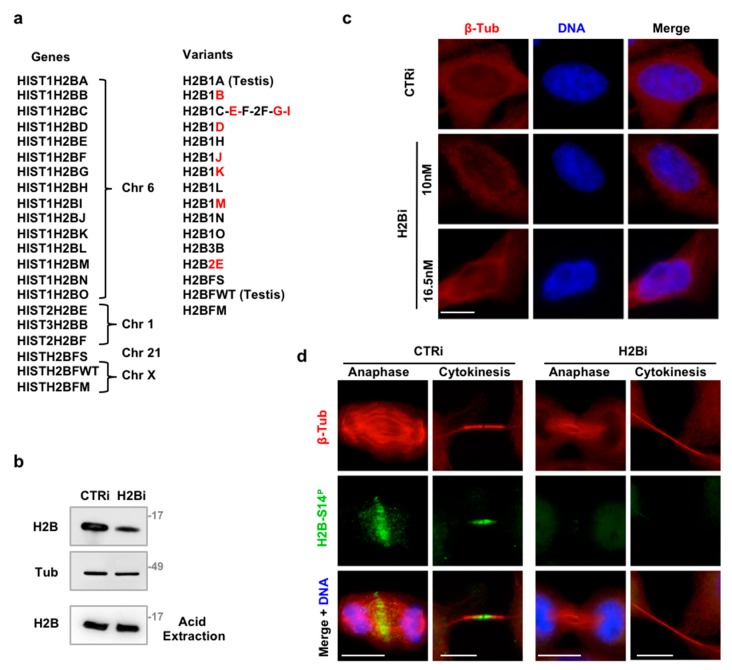
Depletion of ecH2B with siRNAs. (**a**) Schematic representation of H2B isoforms (gene symbol, chromosomal location, and protein name are reported). In red are indicated the isoforms recognized by the nine variant-specific siRNAs. (**b**–**d**) HeLa cells were depleted for histone H2B with a combination of the nine variant-specific siRNAs (H2Bi) or with universal negative control (CTRi). Representative WB for the indicated proteins is shown. Cells were subdivided in two aliquots and cell extracts obtained with lysis buffer and centrifugation to eliminate chromatin or with acid extraction to purify nucleosome histones. (**c**) Representative IF imagines of CTRi and H2Bi HeLa cells—transfected with the indicated molarity of siRNAs—stained with Hoechst (blue) and anti-β-Tubulin Ab (red) to visualize nuclear DNA and cytoplasm. Scale bar is 10 μm. (**d**) Representative IF imagines of CTRi and H2Bi HeLa cells stained with anti-β-Tubulin Ab (red), anti-phospho-H2B-Ser14 Ab (green) and Hoechst (blue) to visualize nuclei. Representative images of cells in anaphase and cytokinesis are shown. At least 30 anaphase and 100 cytokinetic cells form three independent experiments were analyzed. Scale bar is 10 μm.

To investigate the function of ecH2B in cytokinesis, HeLa cells were treated with control siRNA (CTRi) or a mix of nine commercially available H2B siRNAs (H2Bi) that recognize different H2B isoforms (Figure 1a). Transfection with two pulses of 10 nM each siRNA reduced ecH2B expression (Figure 1b) without drastically altering nucleosomal H2B fraction (Figure 1b lowest panel) and chromatin structure (Figure 1c). Next, we analyzed the presence and the subcellular localization of ecH2B in cytokinesis by immunofluorescence (IF) with anti-phosho-H2B-S14 antibody (Ab) that specifically recognizes phosphorylated H2B-S14 (H2B-S14^P^). This Ab was employed to detect specifically ecH2B and avoid immunostaining of nucleosomal H2B, which is not phosphorylated at S14 unless in apoptotic cells [13,46]. To visualize the ICB of cytokinetic cells, microtubules were stained with anti-β-tubulin Ab [29]. CTRi cells showed the expected ecH2B localization at the central spindle, in anaphase, and at the midbody, during cytokinesis (Figure 1d, left panels) [14] while H2Bi cells lacked both types of localization (Figure 1d, right panels). In addition, staining with anti-total H2B Ab that detect H2B at the midbody [13], confirmed the efficacy of the depletion strategy (Supplementary Figure S1).

**Figure 2:**
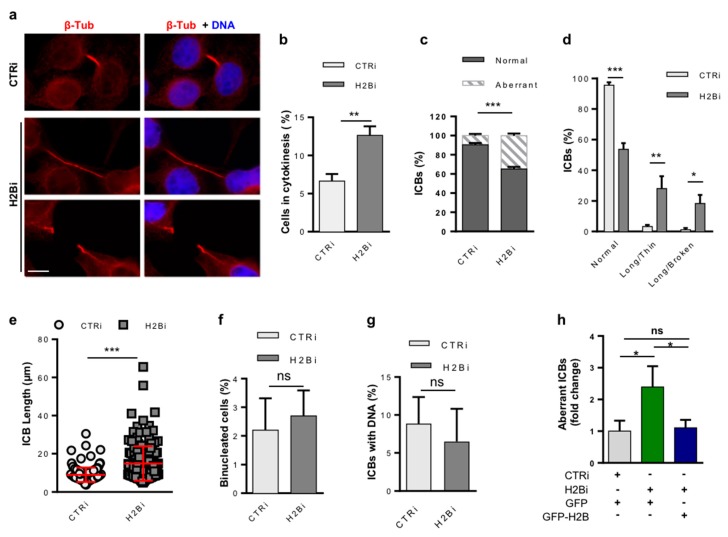
ecH2B depletion in HeLa cells induces cytokinesis defects. Proliferating, asynchronous HeLa cells were depleted for histone H2B, stained for β-Tubulin and DNA as described in Figure 1, and analyzed in panels A to F. (**a**) Representative IF images of cytokinetic cells. Compared to normal cytokinesis in CTRi (upper panels), the presence of long and thin (middle panels) or long and broken (lower panels) ICBs are shown in the H2Bi cells. Scale bar is 10 μm. (**b**) The percentage of cells in cytokinesis was measured by scoring at least 1000 cells per IF sample in four independent experiments. (**c,d**) The relative amount of normal and aberrant ICB was measured by scoring at least 1000 cells per sample and pooling (**c**) or subdividing (**d**) cytokinetic cells based on the defect type. (**e**) The length of each ICB was measured in three independent experiments. (**f**) The presence of cells with two or more nuclei and (**g**) ICBs with DNA (i.e., chromosome bridges, lagging chromosomes) was measured by scoring the same samples described in (b). (**h**) H2Bi and CTRi HeLa cells were transfected with GFP-H2B or GFP-empty vector and stained for β-Tubulin and DNA as above. The amount of long, aberrant ICB was measured by scoring at least 100 GFP-positive cells per sample. Data are reported as mean ± SD. ns *p >* 0.05; * *p* < 0.05; ** *p* < 0.01; *** *p* < 0.001.

To verify whether H2B depletion affects cytokinesis, we counted the number of cytokinetic cells, whose accumulation is an indirect sign of cytokinesis defects [47], and directly assessed the presence of cytokinesis defects by IF for β-tubulin and DNA staining (Figure 2a). Compared to CTRi (*n* = 1000), H2Bi HeLa cells (*n* = 1000) showed a significant accumulation of cytokinetic cells (Figure 2b) with an increased percentage of aberrant phenotypes (Figure 2c), such as long and thin ICBs, several of which were broken but maintained the microtubule long a thin structure, suggesting mechanical rupture of the bridge (Figure 2a,d). To quantify this phenotype, we measured the length of each single, unbroken ICB (CTRi *n* = 151 and H2Bi *n* = 168) and found that, on average, they were twice as long in the H2Bi than in the CTRi cells (Figure 2e). On the same IF samples, we evaluated the presence of binucleated cells, a marker of cytokinesis failure [47] and ICBs with trapped chromatin that would activate the abscission checkpoint [26]. We did not find any accumulation of binucleated cells (Figure 2f) nor of ICBs with DNA (Figure 2g). Furthermore, we did not find evidence of micronuclei in H2Bi cells compared to CTRi, suggesting that ecH2B-depletion does not prevent cleavage or induce accumulation of DNA bridges. Comparable results were obtained by employing immortalized human fibroblasts (Supplementary Figure S2) excluding cell-specific behavior. In addition, overexpression of GFP-H2B in the H2Bi HeLa cells blocked the accumulation of cells with aberrant ICBs, as assessed by evaluating the GFP-positive cells (Figure 2h), excluding off-target effects.

To evaluate whether the ecH2B function in cytokinesis is linked to a specific H2B isoform, we transfected HeLa cells with each single, isoform-specific siRNA (Figure 1a) and measured the percentage of aberrant ICBs (Supplementary Figure S3), as described above. The single siRNAs induced accumulation of cells with aberrant ICBs unless they were not able to deplete ecH2B. These results suggest that cytokinesis role of ecH2B is not played by one specific isoform.

### 3.2. EcH2B is Dispensable for Cytokinesis Factor Recruitment

**Figure 3:**
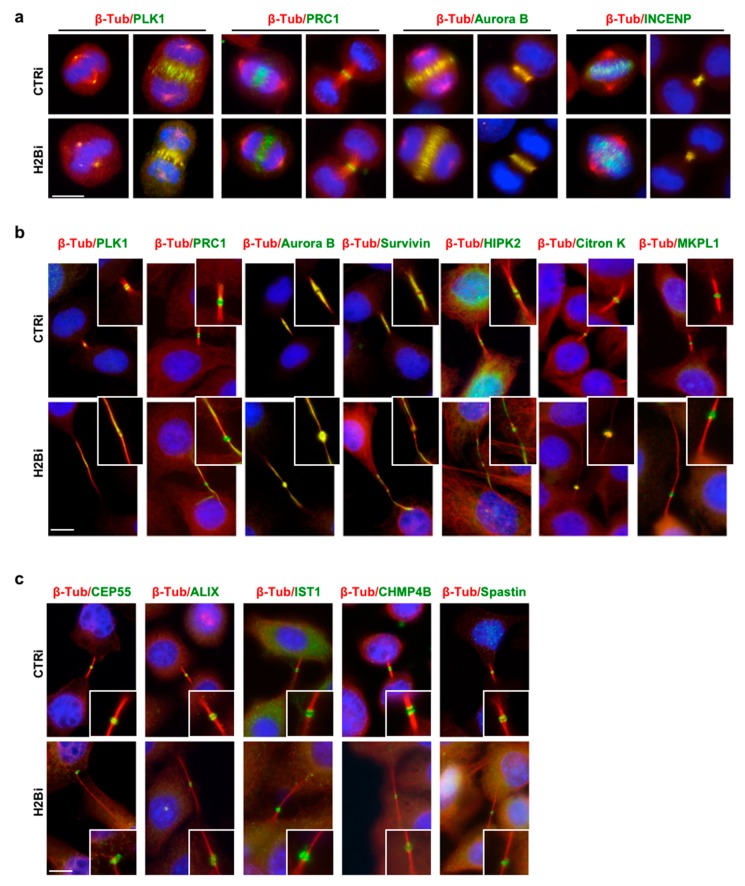
ecH2B depletion does not affect the recruitment of cytokinetic proteins. Proliferating, asynchronous HeLa cells were depleted for histone H2B, as described above, and analyzed by double IF for β-Tubulin (red) and the indicated proteins (green); DNA was marked with Hoechst (Blue). The analyzed proteins are associated with (**a**) central spindle formation and cleavage furrow ingression, (**b**) midbody formation, and (**c**) abscission machinery. Representative images of CTRi and H2Bi HeLa cells are shown. At least 20 metaphases and anaphases in (**a**) and 50 cytokinetic cells in (**b**,**c**) from two independent experiments have been scored. For each of the analyzed proteins, no significant difference was observed in the percentage of positive ICBs that was > 98% in both H2Bi and CTRi cells. Scale bar is 10 μm.

To begin investigating the mechanisms underlying cytokinesis activity of ecH2B, we compared the spatiotemporal localization of a series of structural and functional proteins sequentially recruited during cytokinesis to assure proper cell division [23]. We analyzed the epistasis within ecH2B and these proteins by comparing their presence and localization in CTRi and H2Bi HeLa cells from anaphase to cytokinesis. We used Ab staining to avoid overexpression artifacts [33]. Thus, selection among cytokinesis factors was also dictated by the availability of specific Abs validated for IF (Supplementary Table S2). In early cytokinesis, during central spindle formation and cleavage furrow ingression, no difference between CTRi and H2Bi cells were observed in morphology and immunostaining of the mitotic Polo-like kinase 1 (PLK1), the Protein Required for Cytokinesis 1 (PRC1), Aurora B and Inner centromere protein (INCENP), two components of the Chromosomal Passenger Complex (Figure 3a), suggesting that ecH2B is not involved in the early steps of cytokinesis. Next, we analyzed a series of proteins associated with midbody formation, such as Aurora B, PLK1, mitotic kinesin-like protein 1 (MKPL1), PRC1, Citron kinase, Survivin, and HIPK2. Although their distribution pattern was less sharp compared to CTRi cells, all these proteins were present at the midbody of H2Bi cells (Figure 3b). Finally, we analyzed the main components of the abscission machinery, such as CEP55, the adaptor protein ALIX, the ESCRT-III components CHMP4B and Increased sodium tolerance 1 (IST1 aka CHMP8), and the microtubule-severing enzyme Spastin. As shown in Figure 3c, also these abscission factors, including Spastin, which is the last to be recruited [26,27], were present at the midbody in the H2Bi cells, indicating that ecH2B is dispensable for recruitment of the abscission machinery.

### 3.3. Depletion of ecH2B Delays Abscission

**Figure 4:**
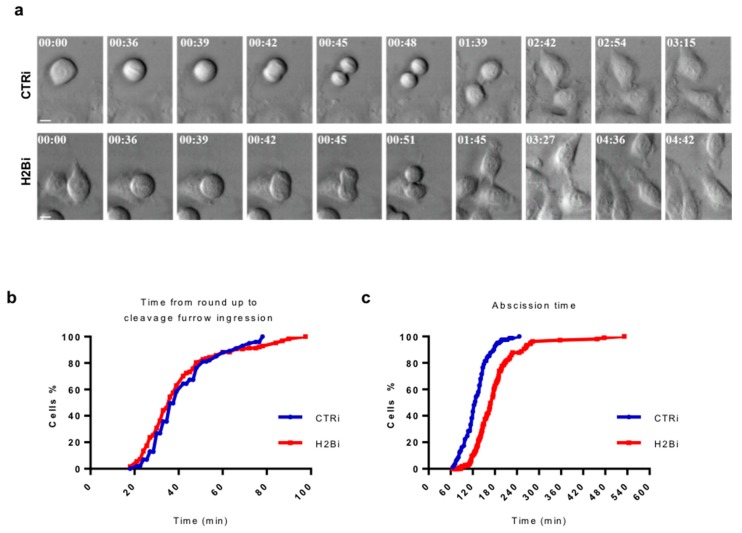
H2B depletion is associated with abscission delay. CTRi and H2Bi HeLa cells were obtained as above and were followed through division by time-lapse microscopy. (**a**) Representative stills from time-lapse recording of HeLa CTRi and H2Bi cells. The time (in hrs:min) since the beginning of the round up is shown. Scale bar is 10 μm. The relative videos are Videos S1 and S2. CTRi cells divide and return mononucleated in about 3 h while H2Bi cells remain connected by intercellular bridge for several hours before abscission occurs. (**b**) The time from round up to cleavage furrow ingression was determined in CTRi HeLa cells (*n* = 150) and in H2Bi (*n* = 100). (**c**) The time from cleavage furrow ingression to abscission (abscission time) was determined in CTRi HeLa cells (*n* = 81) and in H2Bi (*n* = 107). In (**b**) and (**c**), the cumulative percentage of the analyzed cells from four independent transfections is plotted as function of time.

Since no significant alteration in cytokinesis factor recruitment were observed upon H2B depletion, we sought to explain the defect leading to the aberrant telophase phenotype measuring the cytokinesis-time of asynchronous CTRi and H2Bi HeLa cells using time-lapse live-cell imaging (Figure 4a and Videos S1 and S2). No significant difference between the two cell populations were observed in the timing from round up to cleavage ingression, with an average of 40 min for both CTRi (*n* = 150) and H2Bi (*n* = 100; Figure 4b). In contrast, analysis of the abscission time, i.e., the timing from cleavage furrow ingression to physical separation of the two daughter cells, showed a significant delay in the H2Bi cells (182.36 ± 69.5  min in H2Bi (*n* = 107) versus 129.56 ± 35.2  min in CTRi (*n* = 81); *p*  <  0.0001; Figure 4c). In particular, 50% of CTRi cells were severed after 130 min from the cleavage furrow ingression. At the same time, only 15% of the H2Bi cells were severed. This delayed abscission was also associated with the appearance of ICBs that progressively stretched into thin and overextended structures (Video S2), resembling the long and thin intercellular bridges observed in IF (Figure 2a) and, in several cases, persisted for more than 4 hrs. Consistent with the absence of accumulation of binucleated cells observed in static IF conditions (Figure 2f), abscission delay did not associated with regression of the cleavage furrow and fusion of the daughter cells (Video S2 and data not shown). These results show that ecH2B depletion affects the last step of cell division.

### 3.4. H2B Binds and Colocalizes with CHMP4B at the Intercellular Bridge

**Figure 5:**
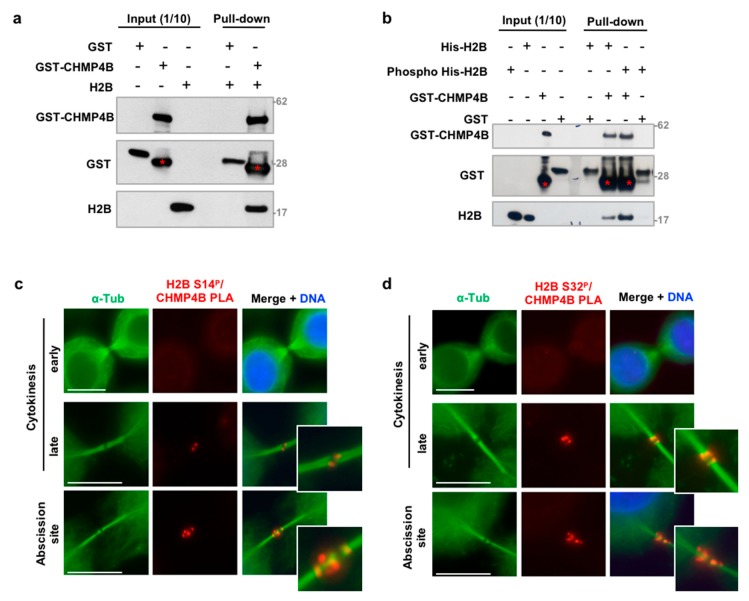
ecH2B binds and colocalizes with CHMP4B at the intercellular bridge. (**a**) GST-pull down was performed to analyze the binding between recombinant GST-CHMP4B and H2B. GST alone was used as negative control. WBs were performed to detect the indicated proteins. Red asterisks (*) indicate GST-CHMP4B sub-products consistently present in the commercial preparation. (**b**) Recombinant His-H2B was phosphorylated at Ser14 by incubation with HIPK2 Kinase Domain (HIPK2 KD) in the presence of cold γ-ATP. GST-pull down was performed to compare the binding between recombinant GST-CHMP4B and His-H2B or His-H2B-S14^P^. GST alone was used as negative control. WBs were performed to detect the indicated proteins. Red asterisks (*) indicate GST-CHMP4B sub-products. (**c,d**) Proliferating, asynchronous HeLa cells were fixed and analyzed by proximity ligation assay (PLA) with anti-CHMP4B Ab and anti-phospho-H2B-Ser14 in two independent experiments (**c**) or anti-phospho-H2B-Ser32 Abs in two other independent experiments (**d**). Representative images of cells in early and late cytokinesis and abscission sites are shown. 30 ICBs per sample were analyzed and overall PLA positivity was observed in 37.7 ± 2.5% of the ICBs. Scale bar is 10 μm.

The most abundant ESCRT-III component CHMP4B and the kinase responsible for H2B phosphorylation at the midbody HIPK2 were previously identified as putative interactors in a yeast two-hybrid screen [48]. To characterize the mechanisms for how H2B depletion affects cytokinesis progression, we asked if an interaction between H2B with the ESCRT-III machinery might exist and be modulated by H2B phosphorylation. GST-pulldown experiments showed that recombinant CHMP4B binds histone H2B (Figure 5a). Since ecH2B contributes to cytokinesis when phosphorylated at S14 by HIPK2 [13], we performed the GST-CHMP4B pulldown experiment with His-H2B phosphorylated by HIPK2 in a cold kinase assay (Supplementary Figure S4). We found that H2B-S14^P^ shows a stronger binding with CHMP4B than the non-phosphorylated H2B (Figure 5b), suggesting a functional relevance of this posttranslational modification in the protein/protein interaction.

Next, we evaluated whether ecH2B and CHMP4B colocalize at the ICB by performing a proximity ligation assay (PLA). To avoid Ab-related nonspecific signals, we employed both anti-phospho-H2B-S14 and anti-phospho-H2B-S32 Abs. No PLA signals were detected with both Abs in early cytokinesis (Figure 5c,d, upper panels) when ecH2B is already present at the ICB but CHMP4B has not yet been recruited [26,27], supporting the specificity of the reaction. In late cytokinesis, when CHMP4B is recruited at the midbody, PLA signals were detected at the dark zone, the typical CHMP4B localization (Figure 5c,d, middle panels). Interestingly, PLA signals were also reproducibly detected at the abscission site, where CHMP4B contributes to the formation of the ESCRT-III helices involved in the physical separation of the two daughter cells (Figure 5c,d, middle and lower panels). Taken together, these results indicate that phosphorylated ecH2B colocalizes with CHMP4B both at the midbody and at the abscission site.

### 3.5. ecH2B Delimits the Abscission Site and Contributes to its Formation

**Figure 6:**
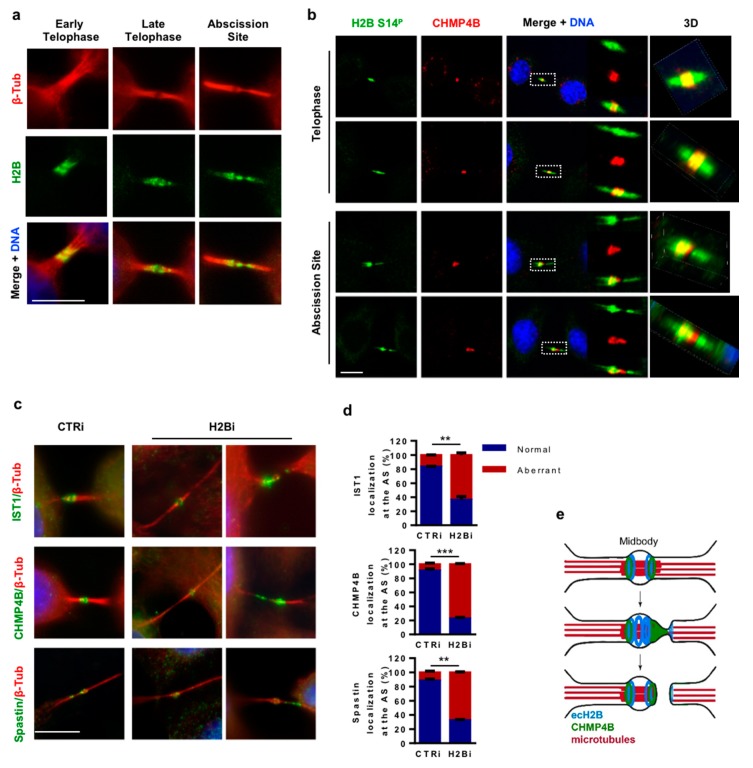
ecH2B contributes to abscission site. (**a**) Proliferating, asynchronous HeLa cells were fixed and analyzed by double IF with anti-β-Tubulin Ab (red) and anti-phospho-H2B-Ser14 Ab (green) in more than five independent experiments. Representative images of early and late cytokinesis and abscission sites are shown. Fifty ICBs per sample were analyzed. Scale bar is 10 μm. (**b**) Proliferating, asynchronous HeLa cells were fixed and analyzed by double IF with anti-phospho-H2B-Ser14 Ab (green) and anti-CHMP4B Ab. Two representative images for late cytokinesis, abscission site and the relative 3D reconstructions are shown. (**c**,**d**) CTRi and H2Bi HeLa cells were obtained as above and analyzed by double IF for β-Tubulin (red) and the indicated proteins (green); DNA was marked with Hoechst (Blue). Representative images (**c**) and quantification (**d**) from three independent experiments of abscission site formation based on the three abscission factors are reported. Data are reported as mean ± SD. ** *p* < 0.01; *** *p* < 0.001. (**e**) Graphical abstract is reported. Microtubules are indicated in red, ecH2B is represented in blue and CHMP4B in green.

To relate H2B dynamics to abscission in fixed HeLa cells, we first co-stained H2B and β-tubulin by double IF and analyzed the behavior of ecH2B in the last steps of cell division. We found that, in early cytokinesis, ecH2B symmetrically localizes at the midbody, in the flanking zone (Figure 6a, left panels). In late cytokinesis, ecH2B began to show an asymmetric distribution toward one of the arms of the intercellular bridge (Figure 6a, middle panels), to end up with a particular localization at the proximal and distal sides of the abscission site (Figure 6a, right panels). Next, we analyzed the relationship between ecH2B and CHMP4B at the midbody and the abscission site by assessing their colocalization by IF. Confocal analyses were performed on double IF images obtained on endogenous ecH2B and CHMP4B with rabbit anti-phospho-H2B-S14 Ab and mouse anti-CHMP4B Ab. We observed that, in telophase, when CHMP4B localizes at the midbody in the flanking zone, ecH2B colocalizes with CHMP4B for its entire distribution and symmetrically protrudes over CHMP4B at both arms of the ICB (Figure 6b, upper panels). Next, when CHMP4B—together with the other components of the ESCRT-III complex—organizes into narrowing cortical spirals toward the abscission site [28,29,30,31,32,33], ecH2B colocalizes with CHMP4B in the cone-like shape of the spirals. Consistently with the ecH2B localization at the flanking sides of the abscission site, we also observed that the CHMP4B/ecH2B colocalization stops at the abscission site, where only CHMP4B is detectable, while ecH2B alone delimits the abscission site at its distal side (Figure 6b, lower panels). Comparable results were obtained by IF of exogenous CHMP4B-Myc with anti-Myc Ab and endogenous H2B (Supplementary Figure S5) excluding Ab-related artifacts.

Due to the particular localization of ecH2B and its relationship with CHMP4B, we asked whether the cell division defects we observed in the H2Bi cells might be related to an impaired formation of the abscission site and subsequent re-localization of the membrane scission and microtubule severing factors. Thus, we compared CTRi and H2Bi HeLa cells in the late stage of abscission for the presence and localization of CHMP4B, IST1, an additional ESCRT-III component whose detection by IF has been employed to resolve ESCRT-III spirals [33], and Spastin. In contrast to CTRi cells, in which a small pool of these proteins accumulated at or toward the abscission site, away from the center of the midbody, in the H2Bi cells this localization is completely missing and CHMP4B, IST1, and Spastin can only be detected at the midbody, despite the long and thin shape of the ICBs, or as multiple dots along one arm of the remaining ICBs (Figure 6c,d and Supplementary Figure S6). These results indicate that in human cells ecH2B contributes to the formation of the abscission site and the localization of the fission machinery.

## 4. Discussion

In the past years, a few histones have been shown to execute extrachromosomal activities that, nonetheless, are related to DNA, such as apoptotic chromatin and viral DNA [8,10]. We previously identified an extrachromosomal localization of histone H2B at the central spindle and the intercellular bridge connecting sibling cells during cytokinesis. This ecH2B localization requires the kinase activity of Aurora B whilst is independent of both DNA and RNA [13,14]. In this work, we provide evidence that the extrachromosomal activity of histone H2B directly contributes to cell division through the generation of the abscission site and the re-localization, from the midbody toward this transient structure, of key fission factors.

In humans there are 16 histone H2B isoforms whose expression and specific functions at the chromatin level are emerging [7]. Thus, we asked whether the cytokinesis activity ecH2B might belong to specific histone H2B variant(s). However, the single variant-specific siRNAs (i.e., H2B.B/D/E/G/K/H/I/J/2E) resulted in the appearance of cytokinetic defects with six out of nine siRNAs suggesting cross-depletion among the different H2B isoforms. Thus, these results do not allow assigning the cytokinesis function of ecH2B to a specific H2B isoform.

Analyses of central spindle and midbody structure and markers in control and H2B-depleted cells suggest that ecH2B is not necessary for the recruitment of proteins involved in central spindle formation, cleavage furrow ingression, midbody formation, and midbody recruitment of the abscission machinery. Each of these subsequent stages hangs on the correct execution of the previous one [18,19,20,47]. Thus, though our analyses were necessarily confined to a partial number of cytokinesis markers, the proper formation of the midbody and the localization at the midbody bulge of CEP55 and the downstream abscission factors ALIX, CHMP4B, IST1, and Spastin—the last member of the abscission machinery to be recruited at the midbody—strongly indicate that ecH2B is dispensable for these cytokinesis stages. Instead, we found significant defects in late midbody maturation, when the fission machinery moves to one or both midbody sides, and H2B-depletion results in the lack of CHMP4B, IST1, and Spastin re-localization at the abscission site. The re-localization of CHMP4B and IST1, two of the key components of the ESCRT-III helices, and Spastin, the enzyme responsible for microtubule severing, are required for the formation of the abscission site and the subsequent fission of the ICB. Depletion of each of these factors has been reported to induce abscission delay and accumulation of long ICBs [33], as we observed in our H2Bi cells, supporting a role for ecH2B in the generation of the abscission site. In agreement with these functional data, we observed that H2B binds CHMP4B in vitro and colocalizes with CHMP4B in vivo, at the flanking zone in early midbodies and toward the abscission site in late midbodies. When the abscission site is formed and clearly visible, the localization of these two proteins ends up with a particular reciprocal distribution, i.e., both CHMP4B and ecH2B at the proximal side of the abscission site, CHMP4B alone at the abscission site, and ecH2B alone at the distal side (Figure 6e). Altogether, these data indicate that ecH2B is required for the proper localization of the fission machinery and the formation of the abscission site.

We previously reported that depletion of HIPK2, the kinase responsible for H2B-S14 phosphorylation at the ICBs, induces a stronger phenotype (i.e., cytokinesis failure and accumulation of binucleated cells) [13] than the one we observed here with depletion of the HIPK2 target, H2B. This divergence can be explained by the recent observation that HIPK2, similarly to other cytokinesis regulating kinases, contributes to cytokinesis by acting on multiple targets. In particular, we observed that, in addition to the absence of H2B-S14 phosphorylation, HIPK2 depletion significantly impairs Spastin localization at the ICB supporting the existence of two parallel mechanisms of action of HIPK2 in cytokinesis [49].

At this point, we cannot discriminate whether ecH2B simply contributes to the abscission site maturation that requires local cytoskeletal remodeling, prior to the assembly of ESCRT-III, or whether it also aids the formation of ESCRT-III helices [39,40,41,42,43,44,45]. CHMP4B is the most abundant ESCRT-III component and its depletion severely affects the structural organization and function of the ESCRT-III spirals at the intercellular bridge of dividing cells [33]. Thus, the physical interaction between ecH2B and CHMP4B might support a role for ecH2B in ESCRT-III filament organization. Moreover, to accommodate microtubules, the membrane neck presented by the ICB is much wider (~1 μm) than others closed by ESCRT-III (for instance, an intraluminal vesicle neck at the multi-vesicular bodies is tens of nm). Therefore, additional structural factors are likely to be required to ensure spatiotemporal coordination of membrane sealing [50]. The following observations support how, in mammals, ecH2B might aid ESCRT-III helices formation and/or constriction by acting as a CHMP4B polymerization factor. First, to exert membrane remodeling activities, ESCRT-III subunits cycle between soluble monomers and higher-order assemblies [51]; this process is controlled by a C-terminal auto-inhibitory domain in ESCRT-III subunits, which folding back onto the protein core domain prevents higher order assembly. Therefore, CHMP4B alone cannot polymerize in vitro unless its C-term is deleted or moved away from the ESCRT-III core to allow for monomer interactions [51,52]. This hypothesis is supported by a particular distribution of negative charge in the C-terminal helices of CHMP4B, which would be amenable to interaction with a predominantly positively charged histone. Second, during apoptosis, histone H2B tails mediate chromatin compaction upon phosphorylation at S14 in vertebrates [53]. In analogy to the proposed mechanism driving chromatin compaction, assembly of H2B-S14^P^ might facilitate CHMP4B polymerization by releasing the auto-inhibitory activity of its C-term. Due to the expanding roles of the ESCRT proteins in membrane remodeling associated with several physiological and pathophysiological processes, such as biogenesis of multi-vesicular bodies, virus budding, and reformation of the nuclear envelope [50,54,55,56,57], it will be interesting to test this hypothesis and verify whether ecH2B or other extrachromosomal histones are the functional partner of the ESCRT-machinery not only in cell division.

## Supplementary Materials

The following are available online at https://www.mdpi.com/2073-4409/8/11/1391/s1, Figure S1: ecH2B depletion in HeLa cells, Figure S2: ecH2B depletion in HFs induces cytokinesis defects comparable to those observed in HeLa cells, Figure S3: Depletion of ecH2B with single variant specific siRNAs, Figure S4: HIPK2-mediated phosphorylation of H2B, Figure S5: Colocalization of H2B-S14^P^ and CHMP4B, Figure S6: ecH2B contributes to abscission site, Table S1: List of commercially available H2B variant-specific stealth RNAi used to perform the RNA interference, Table S2: List of antibodies with the relative IF conditions, Video S1: Time-lapse microscopy of CTRi HeLa cells related to Figure 4, Video S2: Time-lapse microscopy of H2Bi HeLa cells related to Figure 4.
