# Supplementary material for: Extrachromosomal Histone H2B Contributes to the Formation of the Abscission Site for Cell Division
Source: Cells. 2019 Nov 5;8(11):1391. doi: 10.3390/cells8111391 (PMC6912571; doi:10.3390/cells8111391)
Supplement: Supplementary file 1 [file cells-08-01391-s001.zip › Supplementary Data and Videos/LM Supplementary Data.pdf]

Extrachromosomal histone H2B is required for the formation of the abscission site for cell division

Laura Monteonofrio, Davide Valente, Cinzia Rinaldo, and Silvia Soddu

SUPPLEMENTARY DATA

Table of Contents

1) Supplementary Table S1
2) Supplementary Table S2
3) Supplementary Figure S1
4) Supplementary Figure S2
5) Supplementary Figure S3
6) Supplementary Figure S4
7) Supplementary Figure S5
8) Supplementary Figure S6

Supplementary Table S1: List of commercially available H2B variant-specific stealth RNAi used to perform the RNA interference.

Gene	siRNA ID
HIST1H2BB	HSS-142383
HIST1H2BE	HSS-144682
HIST1H2BG	HSS-144677
HIST1H2BI	HSS-144687
HIST1H2BD	HSS-142378
HIST1H2BJ	HSS-144946
HIST1H2BK	HSS-150254
HIST1H2BM	HSS-144679
HIST2H2B2E	HSS-144689

Supplementary Table S2: List of antibodies with the relative IF conditions

Primary Abs	Fixation		Dilution - incubation - temperature	Specie
	**Methanol -20°C / 5 min**	**Formaldehyde RT/ 10 min**		
**ALIX (3A9)** (Santa Cruz Biotechnology)	X		1:100 - 2h - RT	Mouse
**Aurora-B (6/AIM-1)** (BD bioscience)		X	1:100 - 1h - RT	Mouse
**CEP55 (6A10)** (Abnova)		X	1:700 - 2h - RT	Mouse
**CHMP4B (13G12)** (Covalab)	X		1:100 - 2h - RT	Mouse
**CHMP4B** (polyclonal) (Protein Tech)	X		1:100 - 2h - RT	Rabbit
**Citron kinase** (kind gift of Prof. Di Cunto)	X		1:100 - 2h - RT	Mouse
**ECT-2 (C20)** (Santa Cruz Biotechnology)	X		1:100 - 1h - RT	Rabbit
**H2B (EP957Y)** (Abcam)	X		1:100 - 2h - RT	Rabbit
**H2B-Ser14P (D67H2)** (Cell Signaling)	X		1:100 - 2h - RT	Rabbit
**H2B-Ser32P** (polyclonal) (Abcam)	X		1:100 - 2h - RT	Rabbit
**HIPK2 (946)** (self-made, 13)	X		1:100 - O/N - +4°C	Rabbit
**INCENP (58-217)** (Abcam)		X	1:500 - 1h - RT	Mouse
**IST1** (polyclonal) (Protein Tech)	X		1:100 - 2h - RT	Rabbit
**MKLP-1 (4110)** (Santa Cruz Biotechnology)	X		1:100 - 2h - RT	Rabbit
**MYC (9E10)** (Santa Cruz Biotechnology)	X		1:100 - 2h - 37°C	Mouse
**PLK1 (F-8)** (Santa Cruz Biotechnology)		X	1: 100 - 2h - RT	Mouse
**PRC1 (C-1)** (Santa Cruz Biotechnology)	X		1:100 - 2h - RT	Mouse
**Spastin (Sp 3G11/1)** (Santa Cruz Biotechnology)	X		1:100 - 2h - RT	Rabbit
**Survivin (AF886)** (Epitomics)		X	1:100 - 1h - RT	Rabbit
**α-Tubulin-FITC(DM1A)** (Sigma-Aldrich)	X	X	1:200 - 1h - 37°C	Mouse
**β-Tubulin-Cy3(TUB2.1)** (Sigma-Aldrich)	X	X	1:200 - 1h - 37°C	Mouse

Supplementary Figure S1

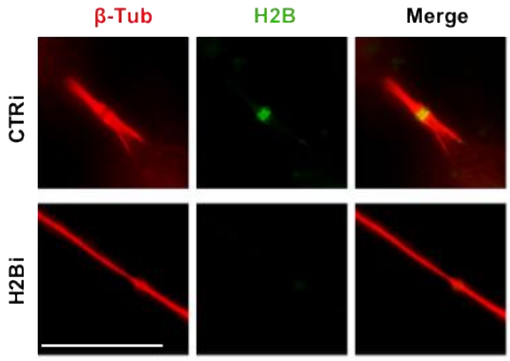

Supplementary Figure S1: ecH2B depletion in HeLa cells. HeLa cells were depleted for histone H2B with a combination of the nine variant-specific siRNAs (H2Bi) or with universal negative control (CTRi) as shown in Figure 1. Representative IF images showing HeLa cells analyzed in cytokinesis for the presence of histone H2B at the ICB. Anti-total H2B Ab (green) and anti-β-Tubulin Ab (red) were used. At least 100 cytokinetic cells from three independent experiments were scored. Scale bar is 10 μm.

Supplementary Figure S2

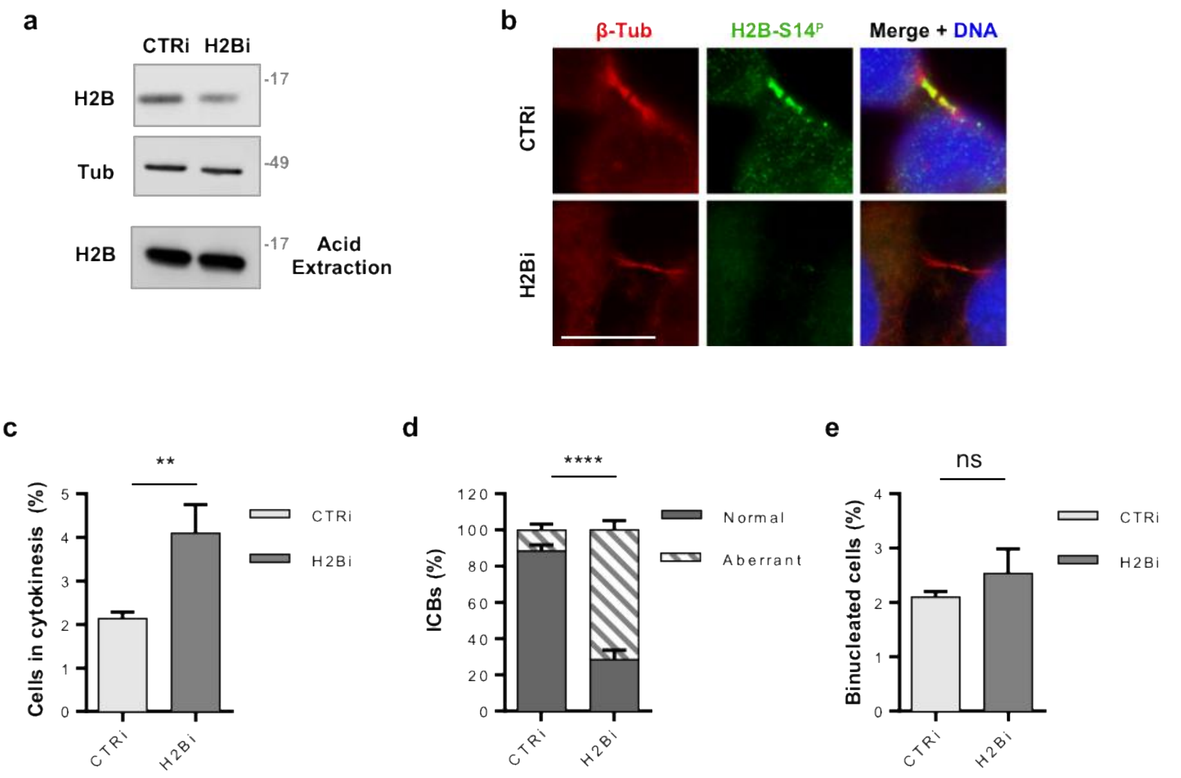

Supplementary Figure S2: ecH2B depletion in HFs induces cytokinesis defects comparable to those observed in HeLa cells. Immortalized human fibroblasts (HFs) were depleted for histone H2B with a combination of the nine variant-specific siRNAs (H2Bi) or with universal negative control (CTRi). (**a**) Representative WB for the indicated protein. (**b**) Representative IF showing HF cells analyzed for the presence of the histone H2B by using the anti-phospho-H2B-S14 Abs. HOECHST (blue) and anti-β-Tubulin Ab (red) was used to visualize nuclear DNA and cytoplasm respectively. Representative images of cells in cytokinesis from at least 100 telophases in three independent experiments are shown Scale bar is 10 µm. H2Bi (n=1000) and CTRi (n=1000) HF cells were analyzed to evaluate the accumulation of cells in cytokinesis (**c**), the presence of aberrant ICBs (**d**) and the presence of binucleated cells (**e**). Data are reported as mean ± SD. ns $p > 0.05$; **$p < 0.01$; *** $p < 0.0001$.

Supplementary Figure S3

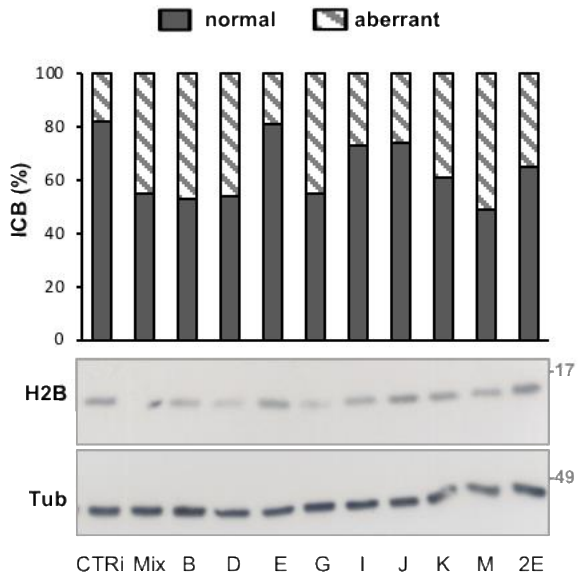

Supplementary Figure S3: Depletion of ecH2B with single variant specific siRNAs. HeLa cells were depleted with each of the indicated isoform-specific, commercially available siRNAs, with a mix of the nine isoforms (Mix), and with the universal negative control (CTRi). There replica experiments have been performed. For each condition, 1,000 cells were analyzed to evaluate the percentage of normal or aberrant ICBs.

Supplementary Figure S4

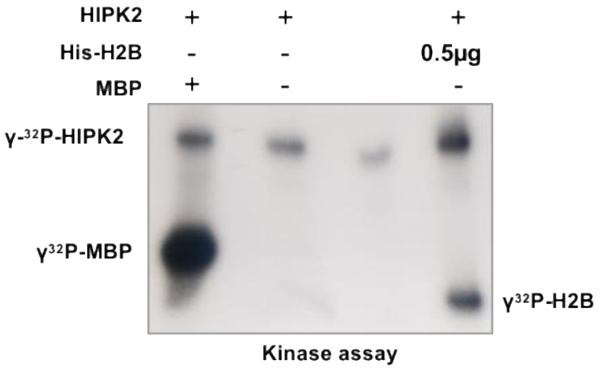

Supplementary Figure S4: HIPK2-mediated phosphorylation of H2B. Recombinant His-H2B was incubated with the HIPK2 Kinase Domain (HIPK2) in the presence of [γ_{32}P]-ATP. Kinase reaction products were resolved by SDS-PAGE and analyzed by autoradiography. A parallel reaction was performed in the presence of cold γ-ATP and employed in the GST-pull down experiments reported in Figure 5b.

Supplementary Figure S5

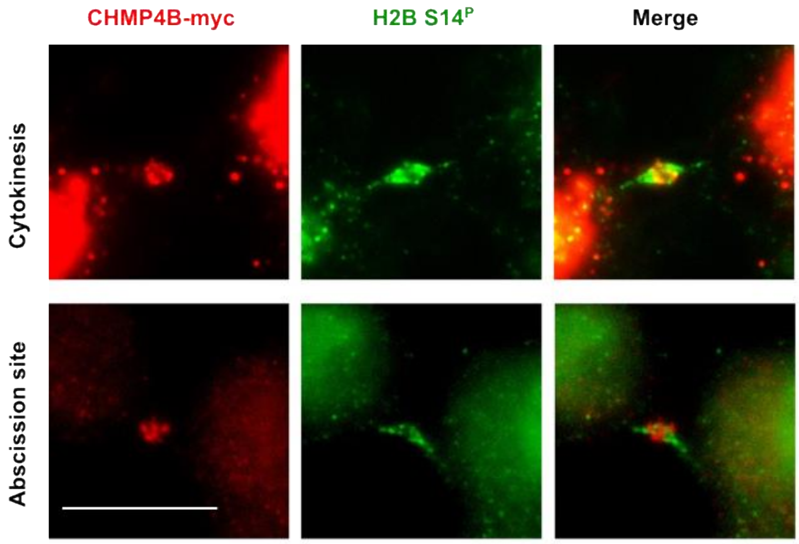

Supplementary Figure S5: Colocalization of H2B-S14P and CHMP4B. HeLa cells were transfected to overexpress CHMP4B-myc protein and co-localization of histone H2B and CMHP4B was analyzed using anti-tag anti-myc Ab (red) and anti-H2B-S14P Ab. Exogenous CHMP4B colocalize with H2B in telophase while at the abscission site CHMP4B/ecH2B colocalization stops at the abscission site, where only CHMP4B is detectable, while ecH2B alone delimits the abscission site at its distal side. Representative image of 20 telophase analyzed were shown. Scale bar is 10 µm

Supplementary Figure S6

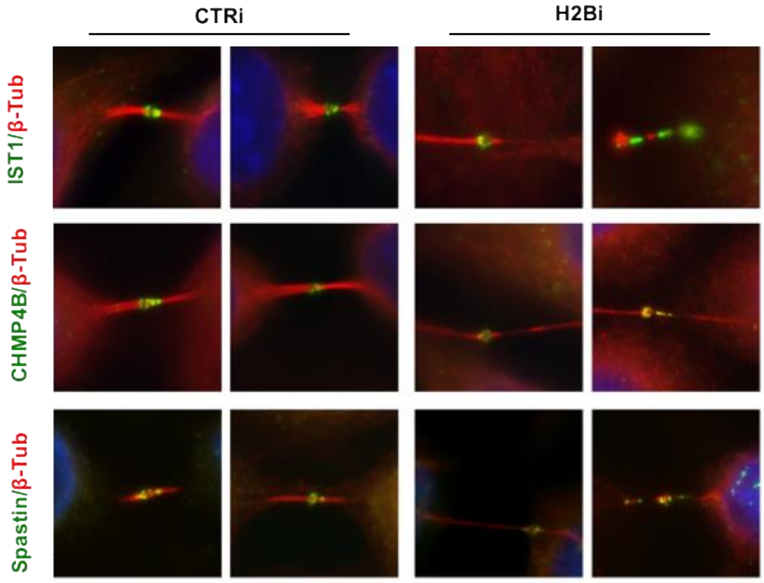

Supplementary Figure S6: ecH2B contributes to abscission site. CTRi and H2Bi HeLa cells were analyzed by double IF for β-Tubulin (red) and the indicated proteins (green) as described in Figure 6c. Additional representative images are reported here.
